# ESTABLISHING A SPANISH FOCUSED ADVANCED CARE PLANNING EDUCATIONAL SESSION FOR LATINA BREAST CANCER SURVIVORS

**DOI:** 10.1093/geroni/igac059.2873

**Published:** 2022-12-20

**Authors:** Moroni Fernandez Cajavilca, Kara Dassel, Gabriela Portugal, Rebecca Utz, Katherine Supiano

**Affiliations:** University of Utah, Bronx, New York, United States; University of Utah, Salt Lake City, Utah, United States; Alliance Community, Murray, Utah, United States; University of Utah, Salt Lake City, Utah, United States; University of Utah, Salt Lake City, Utah, United States

## Abstract

In this exploratory, qualitative study, a focus group was conducted with Latina breast cancer survivors and patients to explore their feedback on how a culturally, sensitive educational session looks like, and whether a Spanish-speaking advanced care planning (ACP) educational session might be able to identify barriers for promoting awareness and discussion of ACP. First, the educational session informed participants on ACP, specifically an advanced directive (AD) and barriers that contribute to decreased rates in ACP among Latino populations. Second, a group interview was facilitated by the project lead using a semi structured interview guide. Eight Latina cancer survivors or patients participated in the educational session. Thematic analysis revealed four themes: 1) Familial Involvement 2) Need for Advanced Care Planning Education 3) Addressing Language and Cultural Barriers and 4) Culturally Sensitive and Informative Resources. A Spanish focused educational session may reduce current barriers that hinder ACP conversations, and may lead to increased rates of participants engaging in AD documentation.

